# A Global Oceanic Dataset of Dissolved Organophosphate Esters

**DOI:** 10.1038/s41597-026-07868-y

**Published:** 2026-07-17

**Authors:** Emily M. Waggoner, Christopher J. Frost, Solange Duhamel

**Affiliations:** 1 Department of Molecular and Cellular Biology, University of Arizona, Tucson, Arizona 85721 USA; 2 BIO5 Institute, University of Arizona, Tucson, Arizona 85721 USA

## Abstract

Organophosphate esters (OPEs) are synthetic compounds widely used as plasticizers and flame retardants in consumer and industrial products. Their increasing detection in marine environments worldwide, coupled with evidence of persistence, bioaccumulation, and toxicity, has raised concerns about their long-term ecological impacts. Given the rapid expansion of oceanic OPE measurements, it is timely to synthesize existing observations and provide a consolidated view of where and which OPEs have been measured to date. Here we present the oceanic OrganoPhosphate Ester dataset (oceanOPEv2025), which compiles 760 seawater measurements from 26 studies conducted between 2014 and 2025. This dataset represents a comprehensive synthesis of OPE concentrations across global ocean basins and seas, offering compiled data on detection frequencies, concentration ranges, and spatial distributions for 24 OPEs. Among the potential applications of oceanOPEv2025, we highlight three priorities for advancing our understanding of OPE transport and fate in marine systems: expanding compound coverage to include high-production OPEs, extending spatial sampling to undersampled basins, and increasing vertical profiling to characterize subsurface distributions. The dataset is publicly accessible with comprehensive metadata, enabling integration with oceanographic databases and facilitating research on the global-scale distribution of these emerging contaminants.

## Background & Summary

**Fig. 1:**
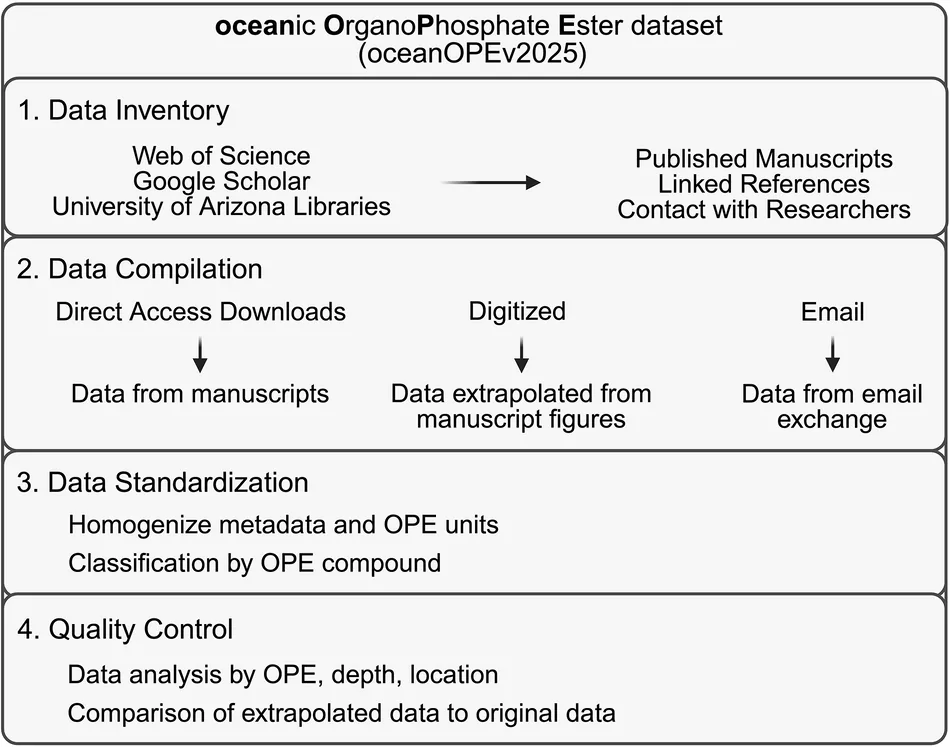
oceanic OrganoPhosphate Ester dataset (oceanOPEv2025) workflow.

**Fig. 2:**
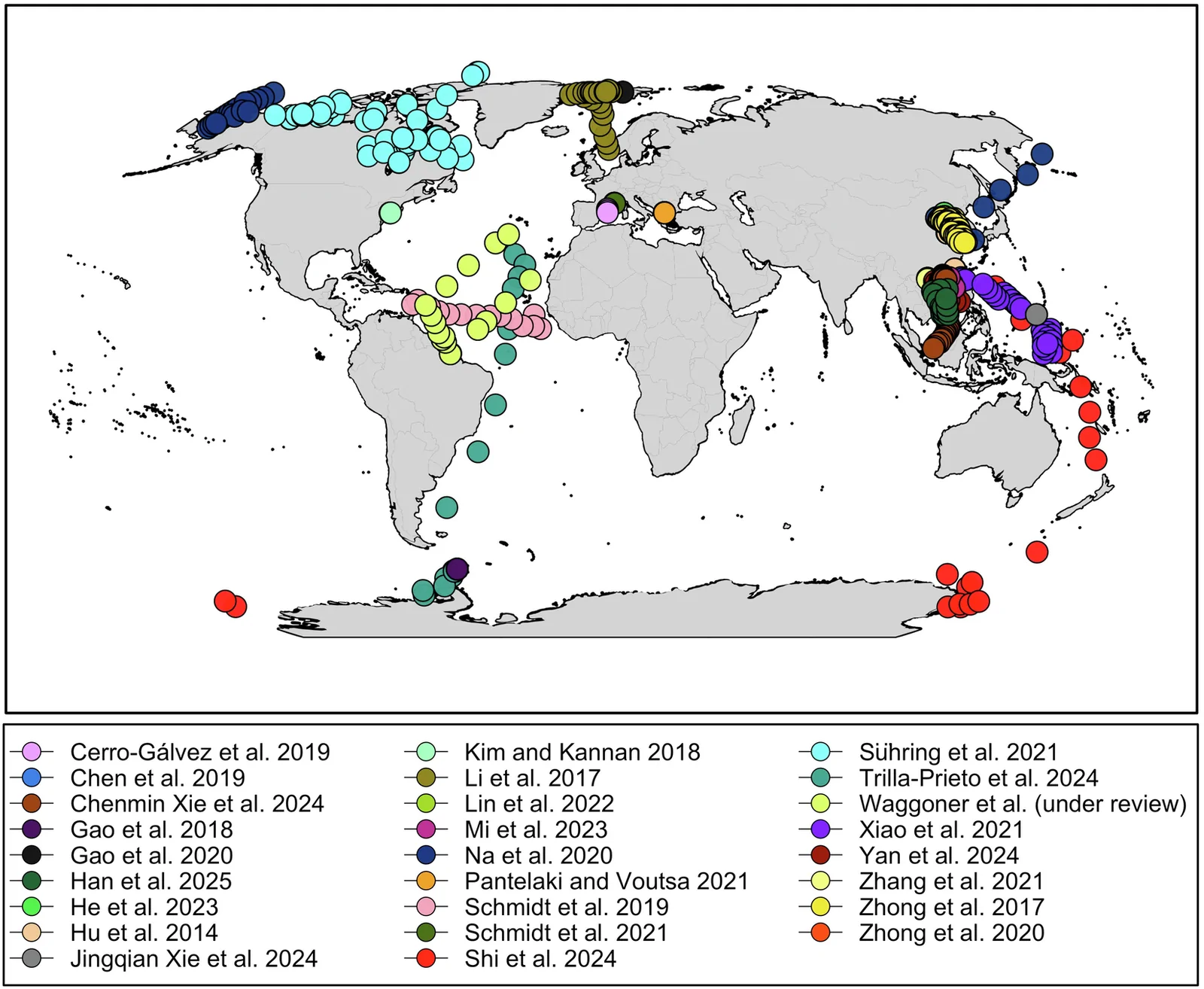
Spatial distribution of OPE measurements included in oceanOPEv2025. Twenty-six studies (color-coded) measuring organophosphate esters (OPEs) in oceanic waters are represented by circles.

Organophosphate esters (OPEs) are a diverse class of synthetic compounds widely used as plasticizers and flame retardants^1–4^, with global production increasing markedly over recent decades^2^. As their prevalence in consumer and industrial products has expanded, so has concern over their biological effects, including disruptions to the endocrine, neurological, developmental, and reproductive systems^5–9^. These health concerns, coupled with physiochemical properties that allow environmental mobility^5,7^, have drawn the attention of the ocean sciences community^6^. OPEs have now been reported in seawater, marine sediments, and marine biota (see review by Xie *et al*.)^6^. This growing body of work provides an opportunity to organize and consolidate existing oceanic OPE measurements. In support of this effort, we compiled the oceanic OrganoPhosphate Ester dataset (oceanOPEv2025)^10^, synthesizing 760 seawater measurements collected from 26 studies between 2014 and 2025. The data compilation and quality control workflow are outlined in Fig. 1. oceanOPEv2025 integrates concentration data for 24 OPEs (details included in the dataset file under ‘OPE_properties’) with sampling metadata (e.g. date, location, depth) and methodological details (e.g. blank correction, recovery adjustment), establishing a comprehensive compilation of oceanic OPE measurements (Fig. 2). Together, oceanOPEv2025 serves as a flexible community resource for assessing the scope of existing oceanic OPE measurements and for supporting future efforts aimed at improving the characterization of oceanic OPEs.

**Fig. 3:**
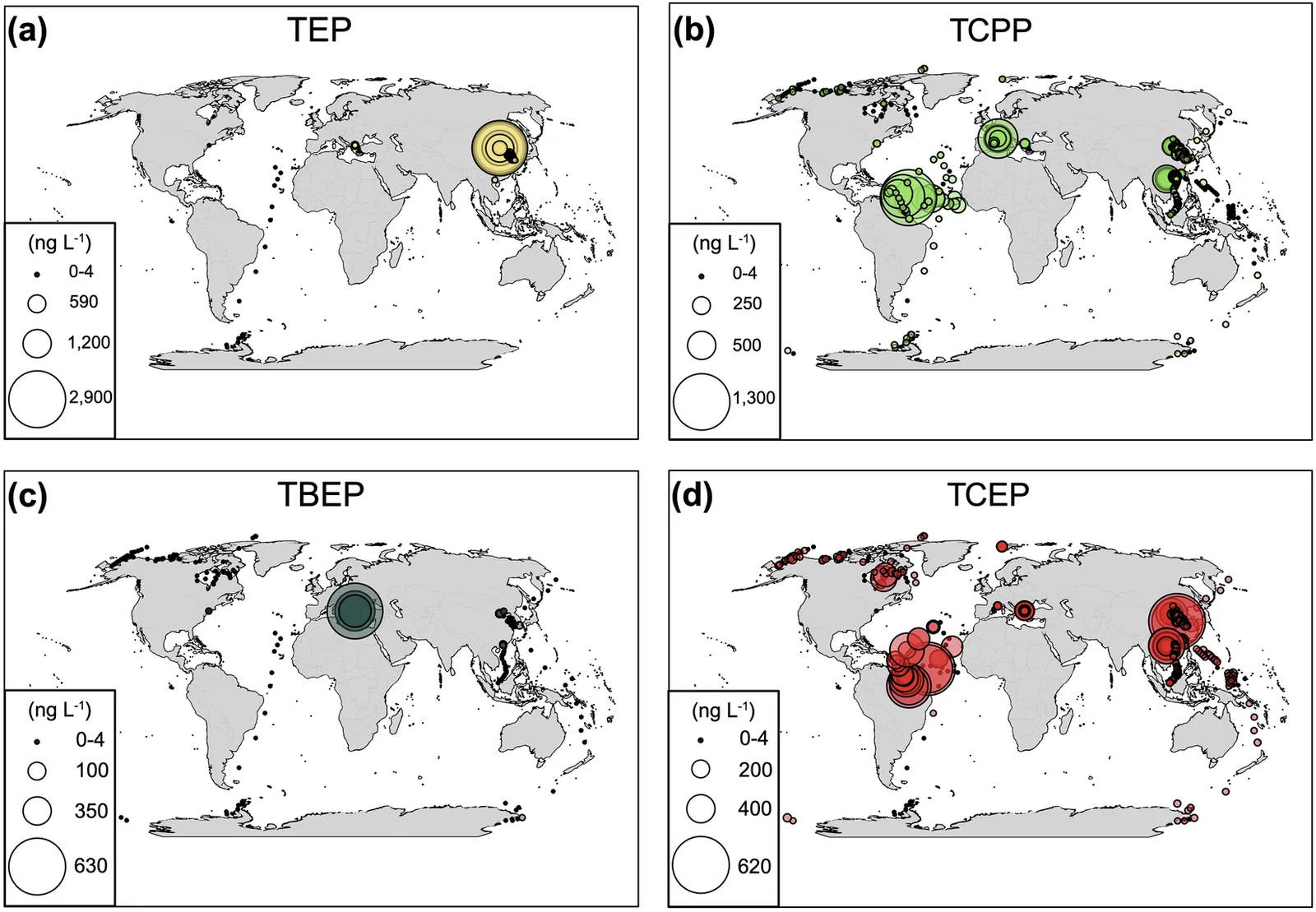
Global distribution of the four OPEs with the highest measured concentrations. Surface water (0–20 m) concentrations (ng L^−1^) of organophosphate esters (OPEs) are displayed for: (**a**) triethyl phosphate (TEP), (**b**) tris (1-chloro-2-propyl) phosphate (TCPP), (**c**) tris (2-butoxyethyl) phosphate (TBEP), and (**d**) tris (2-chloroethyl) phosphate (TCEP), which represent the four OPEs with the highest detected concentrations included in oceanOPEv2025. Data points are represented by circles.

**Fig. 4:**
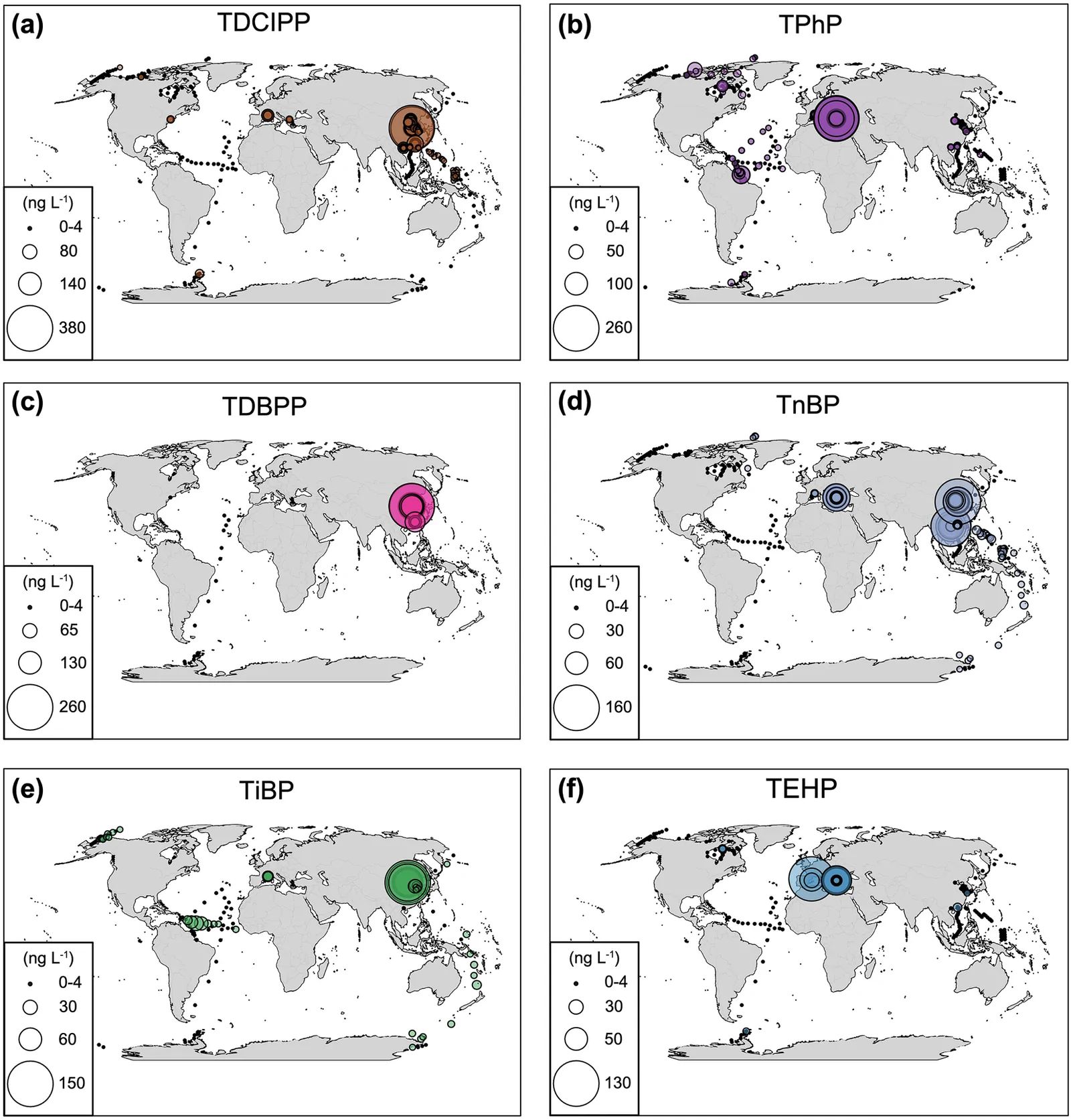
Global distribution of OPEs with the 5^th^-10^th^ highest measured concentrations. Surface water (0–20 m) concentrations (ng L^−1^) of organophosphate esters (OPEs) are displayed for: (**a**) tris (1,3-dichloro-2-propyl) phosphate– TDCIPP, (**b**) triphenyl phosphate– TPhP, (**c**) tris (2,3-dibromopropyl) phosphate– TDBPP, (**d**) tri-n-butyl phosphate– TnBP, (**e**) tri-isobutyl phosphate– TiBP, and (f) tris (2-ethylhexyl) phosphate– TEHP, which represent the 5th to 10th highest detected OPE concentrations in oceanOPEv2025. Data points are represented by circles.

**Fig. 5:**
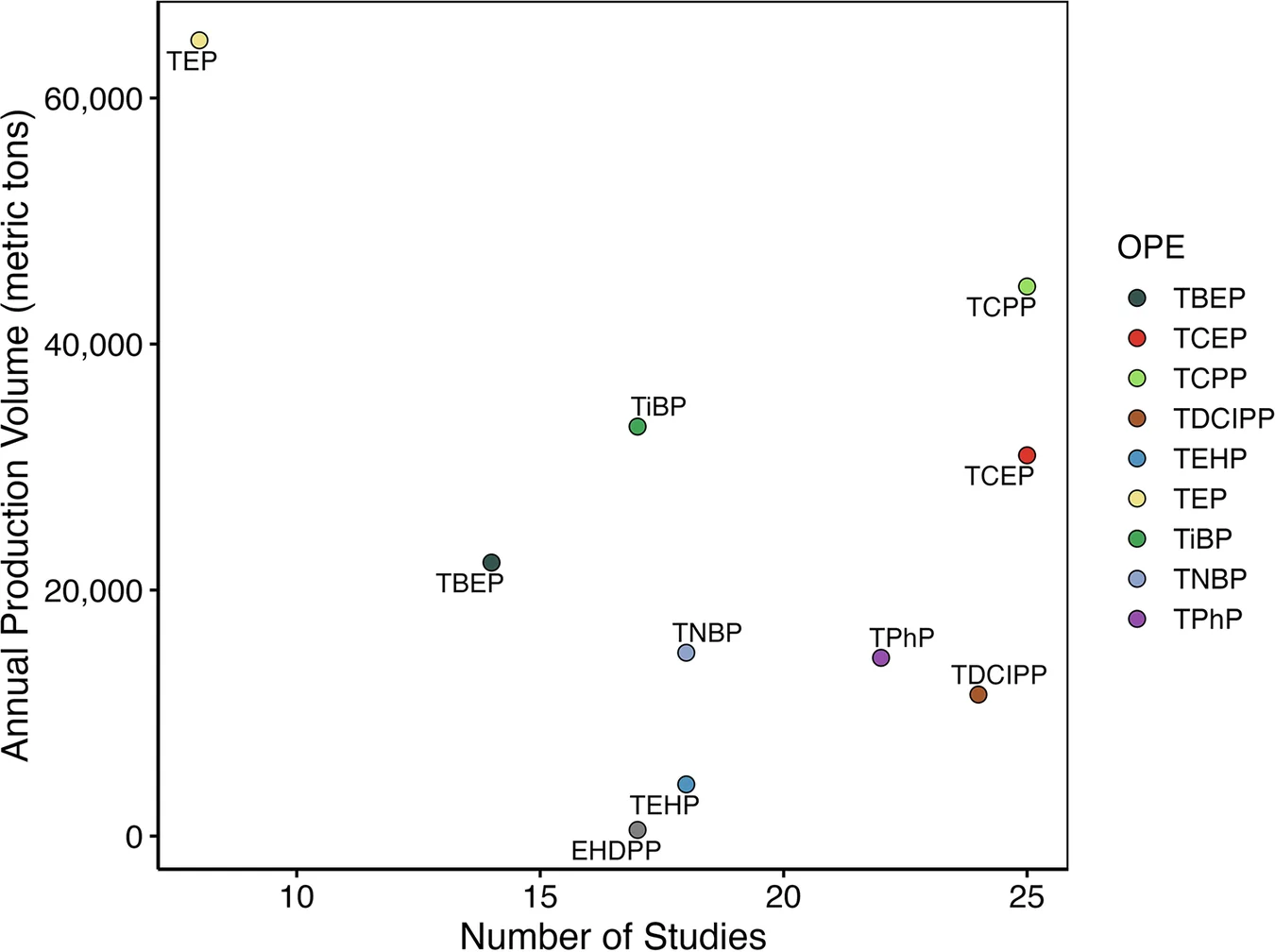
Annual production volume related to the oceanOPEv2025 study frequency. The organophosphate esters (OPEs) annual production volume reported in Huang *et al*.^2^ is displayed on the y-axis as metric tons^2^. The number of studies included in oceanOPEv2025 that target each OPE is displayed on the x-axis.

**Fig. 6:**
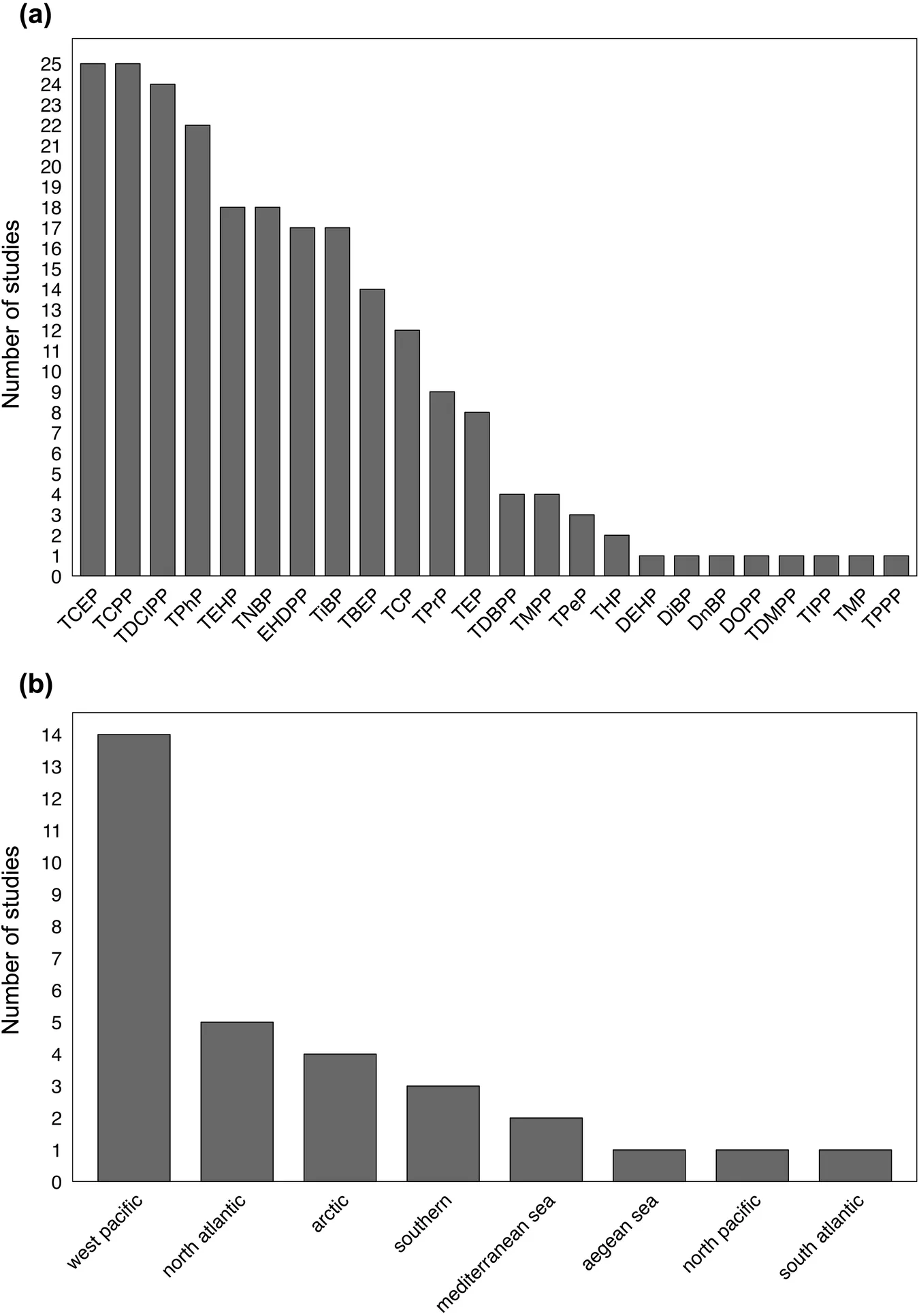
oceanOPEv2025 study frequency. (**a**) Number of studies reporting each of the 24 organophosphate esters (OPEs), and (**b**) Number of studies by oceanic region, ordered by decreasing frequency. Full OPE names are listed in the dataset under ‘OPE_properties’.

The compiled dataset^10^ gives insight into how oceanic OPE research has been approached to date. Using the dataset as a framework, we discuss three potential priority areas for future sampling efforts: compound selection, spatial sampling distribution, and vertical profiling coverage. We first consider OPE compound selection across oceanic OPE studies. Current research efforts concentrate on a limited number of OPE compounds, despite their diverse molecular structures and physiochemical properties. Structurally, OPEs are characterized by a central phosphate group with aryl- or alkyl- sidechains, and can be halogenated^4^. They can be broadly categorized as tri-esters, di-esters, and mono-esters, depending on the number of organic substituents attached to the phosphate group^2^. Tri-esters dominate industrial production and are correspondingly well represented in oceanOPEv2025^10^, comprising 19 out of the 24 compounds (see ‘OPE_properties’ in the dataset file). Among these, 11 tri-esters exhibit the highest global observed concentrations in oceanOPEv2025^10^ (Figs. 3, 4). The four tri-esters with the highest observed concentrations, triethyl phosphate (TEP, 2,938 ng L^−1^), tris (1-chloro-2-propyl) phosphate (TCPP, 1,300 ng L^−1^), tris (2-chloroethyl) phosphate (TCEP, 618 ng L^−1^), and tris (2-butoxyethyl) phosphate (TBEP, 630 ng L^−1^) (Fig. 3), are also among the dominant OPE compounds in global industrial production^1,5,11^. All four are classified as high-production-volume chemicals under the European Registration, Evaluation, Authorization and Restriction of Chemicals framework^12^, with recent estimates placing their annual production volumes (APV) at approximately 65,000, 45,000, 31,000, and 22,000 metric tons, respectively^2^. However, measurement frequencies do not align with production volumes. Despite having the highest and third-highest APVs among the four compounds, TEP and TBEP were included in only 54% and 31% of the compiled studies, respectively, whereas TCEP and TCPP were targeted in 96% of studies (Figs. 5, 6a). Nevertheless, TEP and TBEP exhibited high environmental concentrations, suggesting that broader analytical coverage of these OPEs is needed to better assess the full magnitude of oceanic OPEs.

Di-esters are particularly understudied. Although they are typically formed through the degradation of tri-esters in the environment^11^, di-esters can also originate directly from industrial products and impurities^13^. Di-esters also exhibit potential human health and ecological implications. For instance, Liang *et al*. (2024) and references therein note that some di-esters may exhibit greater endocrine-disrupting potential than their tri-ester counterparts, calling for their broader inclusion in environmental sampling efforts^11^. Yet only 5 of the 24 OPEs in oceanOPEv2025^10^ are di-esters: di-isobutyl phosphate (DiBP), di (2-ethylhexyl) phosphate (DEHP), dioctyl phenyl phosphate (DOPP), 2-ethylhexyl diphenyl phosphate (EHDPP), and di-n-butyl phosphate (DnBP), despite at least 11 di-esters (plus isomers) being environmentally relevant^11^. Additionally, when ranking the maximum observed concentrations for each OPE in oceanOPEv2025^10^, the five di-esters rank as the 12^th^—15^th^, and 22^nd^ highest observed concentrations (see ‘OPE_properties’ in the dataset file). This highlights their environmental prevalence and the need to better understand their distribution, persistence, and potential marine ecological implications.

In addition to compound selection, oceanOPEv2025^10^ includes a basin-scale perspective on where oceanic OPE measurements have been collected to date (Fig. 2). Within oceanOPEv2025^10^, fourteen studies were conducted in the Western Pacific Ocean, most of which were in the Yellow Sea, Bohai Sea, and South China Sea. The Atlantic Ocean accounts for six studies, followed by the Arctic Ocean (four), the Southern Ocean (three), the Mediterranean Sea (two), and the North Pacific Ocean and Aegean Sea (one each) (Fig. 6b). Notably, the Indian Ocean is the only major oceanic basin where concentrations have not been reported, as well as extensive areas in the Pacific and South Atlantic Oceans. Expanding measurements across basins would improve our understanding, and prediction of, oceanic OPE distribution and transport.

**Fig. 7:**
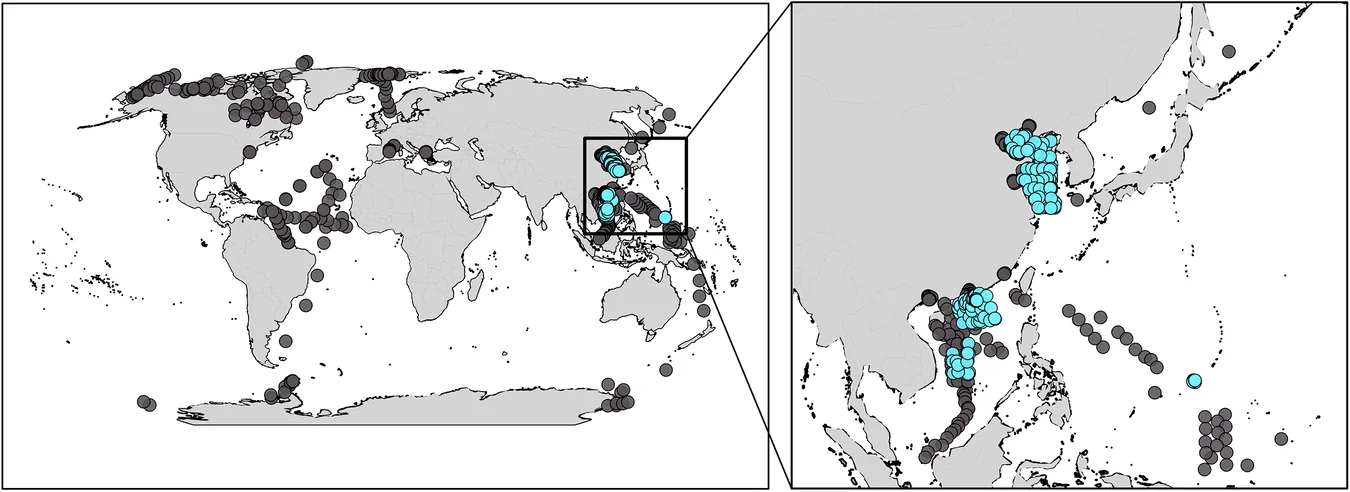
oceanOPEv2025 OPE vertical profiles. The circles represent the latitude and longitude coordinates of study stations sampled in surface waters (0–20 meters; gray circles) and subsurface waters (>20 meters; blue circles)^14–19^ for organophosphate esters (OPEs).

The vertical sampling of OPEs is similarly limited. In oceanOPEv2025^10^, most studies (20 of 26) collected samples from only the surface waters, within the upper 20-meters of the water column (Fig. 7). Only six studies included vertical profiles^14–19^, all conducted in the Western Pacific Ocean (Fig. 7). In the Bohai and Yellow Seas, vertical profiles ranged from the surface to 77-meters^15,18^, while in the South China Sea, sampling extended to 3,695-meters^14,16,19^. The deepest reported sampling occurred in the Mariana Trench, where OPEs were measured from the surface to 11,000-meters^17^, demonstrating that OPEs can be detectable across the water column. Yet fundamental questions remain unresolved: what processes transport OPEs to depth? Do concentrations vary predictably with depth, or do subsurface maxima occur? How do vertical distributions vary with OPE physicochemical properties? Do deep-water OPEs influence biogeochemical cycling or benthic ecosystems? Without expanded vertical profiling across diverse oceanographic regions, we cannot characterize the three-dimensional distribution of oceanic OPEs or assess their impacts on marine environments.

Together, oceanOPEv2025^10^ provides a comprehensive compilation of oceanic OPE measurements. The consolidated view offered by oceanOPEv2025^10^ facilitates the examination of topics such as the compound coverage relative to compound diversity and production, basin-scale patterns in oceanic sampling, and vertical distributions. By integrating disparate measurements into a unified framework, oceanOPEv2025^10^ enables the marine community to move from localized case studies toward global-scale assessment of these emerging contaminants. The dataset is publicly accessible with a permanent DOI and will be updated annually as new measurements become available to maintain its utility as a living resource for OPE oceanic research.

## Methods

We conducted a comprehensive literature search to identify peer-reviewed studies reporting oceanic OPE concentrations. The search was performed between January 2025 and January 2026 across multiple academic service platforms: The Web of Science, Google Scholar, University of Arizona Libraries. Search keywords were ‘organophosphate ester’; ‘organophosphate ester seawater’; ‘OPE’; ‘OPE in oceans’; ‘triphenyl phosphate’; ‘tris chloroethyl phosphate’; ‘organophosphorus flame retardants’; ‘organophosphorus esters’; ‘tri-ester OPE’; ‘di-ester OPE’^20,21^. We excluded organophosphorus compounds with direct carbon-phosphorus bonds (e.g., triphenyl phosphine oxide, TPPO^22–24^) as these are structurally distinct from organophosphate esters^22–24^. OPEs TCPP and tricresyl phosphate (TCP), which have various isomers, were combined under TCPP and TCP, respectively. The final compilation includes 24 OPEs from 25 published studies and one manuscript currently under peer review, yielding 760 data entries.

OPE concentration data and associated metadata were extracted from published sources using multiple approaches. When values were explicitly listed in manuscript text or tables, we transcribed them directly. When concentrations were presented only in figures, we digitized values using WebPlotDigitizer version 4.6 software (https://automeris.io/WebPlotDigitizer). For studies where neither tabular data nor clear figures were available, we contacted authors directly to request data; two studies provided data through this approach^20,21^. Geographic coordinates were extracted from published maps, tables, or supplementary information. When exact coordinates were not provided, we estimated them using WebPlotDigitizer, using the plot scale bars as a reference. OPE concentration units are often reported in either pg L^−1^ or ng L^−1^. All concentrations in the dataset were converted into ng L^−1^.

The 26 studies used diverse sampling approaches reflecting different research objectives and oceanographic settings (Table 1). Discrete water sampling was most common, using stainless-steel collectors^8,16,25–29^, Niskin bottles mounted on a conductivity-temperature-depth sampling device^15–17,19,20,22^ (31% and 23% of oceanOPEv2025^10^ studies, respectively), or glass bottles (19%)^21,30–33^. Additional sampling devices included grab samplers^20,34^ and polypropylene bottles^35^. One study used glass plate samplers to specifically target the sea surface microlayer, where organic contaminants can accumulate^23^. Research vessel intake/underway systems, enabling surface water collection without stopping the ship, accounted for 11% of the studies included in oceanOPEv2025^14,36,37^. Two studies used passive sampling techniques to measure OPEs over time, a method particularly useful in remote or low-concentration environments like the Southern Ocean^38,39^. They used triolein-embedded cellulose acetate membrane (TECAM) and hydrophilic-lipophilic balance sorbent-embedded cellulose acetate membrane (HECAM) samplers to measure the time-weighted average concentration over the deployment period^38,39^.

**Table 1:** Distribution of sampling methods across studies included in oceanOPEv2025.

Sampling Method	Number of Studies	Percentage	Sample Type
Stainless-steel collectors	8^8,16,25–29^	31%	Discrete – surface
Niskin bottles (CTD rosette)	6^15–17,19,20,22^	23%	Discrete – surface & profiling
Glass bottles	5^21,30–33^	19%	Discrete – surface
Underway ship intake	3^14,36,37^	11%	Continuous – surface
Grab samplers	2^20,34^	8%	Discrete – surface
Passive samplers (TECAM/HECAM)	2^38,39^	8%	Time-integrated
Glass plate samplers	1^23^	4%	Surface microlayer
Polypropylene bottles	1^35^	4%	Discrete – surface

Across the compiled studies^10^, OPE quantification followed similar workflows despite some methodological variation. Most studies used solid-phase extraction (SPE) to isolate and preconcentrate dissolved OPEs from seawater samples prior to instrumental analysis (e.g. refs. ^14,18,40^). Common SPE cartridges included Oasis HLB and Bond Elut ENV, which retain OPEs while removing matrix interferences. Analytical detection was typically achieved through gas chromatography coupled to mass spectrometry (GC-MS or GC-MS/MS) or liquid chromatography–tandem mass spectrometry (LC-MS/MS) (e.g. refs. ^11,41^). Method development increasingly incorporates isotopically labeled internal standards (e.g. D_27_-TiBP, D_18_-TCPP, D_15_-TDCP, and D_15_-TPhP) to improve quantification accuracy and correct for matrix effects and recovery losses during sample processing, which was consistently included in the studies of this dataset (e.g. refs. ^23,25^).

Quality control methods, including blank and recovery corrections, are applied to sample datasets before interpretation. Across the 26 studies, blank-handling procedures varied but fall into several widely used categories. Thirteen^14,15,17,22,24,29,32,33,35,36,38,39^ studies incorporated procedural or field blanks into the calculation of method detection or quantification limits (e.g. mean blank concentration plus 3σ or 10σ; significantly lower than limit of detection) and reported concentrations without explicit blank subtraction, with values below these thresholds treated as non-detects (see ‘data’ in the dataset file). In contrast, ten^16,18–21,23,25–28^ studies applied explicit blank correction by subtracting mean blank concentrations and subsequently applied method detection limits or significance-based criteria to ensure concentrations remain distinguishable from background contamination. Two^30,31^ studies adopted compound-specific approaches, applying blank correction only for OPEs detected in blanks while using signal-to-noise-based detection limits for others. One^37^ study used signal-to-noise-based detection limits. Together, these approaches reflect differing assumptions about the origin, consistency, and analytical relevance of OPE background contamination. Where possible, oceanOPEv2025^10^ records whether the final OPE concentrations are blank corrected and the method used in data analysis. Sample concentrations can also be adjusted based on recovery testing, either of a known OPE concentration or based on the isotopically labeled standards. oceanOPEv2025^10^ records whether concentrations were adjusted for recoveries: 11% of studies applied recovery corrections, 61% did not, and 27% did not report this information.

## Data Records

The oceanic OrganoPhosphate Ester dataset (oceanOPEv2025)^10^ is an Excel Workbook (xlsx) that can be accessed on Figshare (https://doi.org/10.25422/azu.data.30560015). All data records in this manuscript correspond to Version 1 (2025) of oceanOPEv2025. The file contains five sheets named ‘README’, ‘data’, ‘OPE_properties’, ‘data_source’, and ‘analytical_limits’. The first sheet, ‘README’, includes descriptions of each column of the ‘data’ sheet and ‘analytical_limits’ sheet. The sampling details and OPE concentrations are listed on the second sheet titled ‘data’. The third sheet, ‘OPE_properties’, defines the 24 OPEs in the dataset (full compound name, abbreviation, chemical formula, molecular weight, and molecular structure). In the fourth sheet, ‘data_source’, DOI’s for each of the published articles included in oceanOPEv2025 are outlined. Lastly, sheet five, ‘analytical_limits’, includes the type of analytical limits that each study reported, including limits of detection (LOD, MDL, IDL) and limits of quantification (LOQ, MQL, IQL). ‘data’ includes the columns below, in addition to the concentrations of the 24 OPEs. The dataset will be updated by the authors annually.

dataset_number

date_added

data_source

year_published

station_code

sampling_date

sampling_year

sampling_month

ocean_basin

sea

latitude

longitude

sampling_platform

cruise_id

sampling_depth

sampling_volume

sampling_type

recovery_corrected

blank_corrected

blank_type

blank_handling

measurement_unit

TPhP……

## Technical Validation

WebPlotDigitizer is used in estimating values from published figures and has been used in other dataset publications (e.g. Su *et al*.)^42^. The accuracy of WebPlotDigitizer was examined with manuscripts that included both figures and data, allowing for a comparison between the actual and predicted values. For concentrations <100 ng L^−1^, recovery of digitized values ranged from 100–120% compared to reported values and for concentrations >100 ng L^−1^, recovery ranged from 94–100%. As such, researchers should keep in mind that concentrations <100 ng L^−1^ may be overestimated (i.e., 120% recovery = 90 ng L^−1^ reported here, as opposed to 75 ng L^−1^ in the reported study).

## Usage Notes

Users should note that the reported concentrations are influenced by study-specific sampling techniques and the application of blank and recovery corrections. Information on sampling methods and data corrections is included in oceanOPEv2025, where possible, to help users select data appropriately. These methodological differences should be carefully considered when analyzing or comparing data within oceanOPEv2025^10^.

## Data Availability

The oceanic OrganoPhosphate Ester dataset (oceanOPEv2025)^10^ is an Excel Workbook (xlsx) that can be publicly accessed on Figshare (https://doi.org/10.25422/azu.data.30560015). The dataset will be updated annually by the authors.

## Code availability

No custom code has been used to produce this database. RStudio (version 4.3.3) was used for visualization of the data using the ‘oce’, ‘ocedata’, and ‘ggplot2’ packages.
